# The quality of life of people with multiple sclerosis in Portugal

**DOI:** 10.15649/cuidarte.2841

**Published:** 2024-08-08

**Authors:** Rosa Martins, Sara Gomes, Patricia Vicente, Nélia Carvalho, Susana Batista

**Affiliations:** 1 Escola Superior de Saúde do Instituto Politécnico de Viseu, Portugal. E-mail: rmartins.víseu@gmail.com Instituto Politécnico de Viseu Escola Superior de Saúde Instituto Politécnico de Viseu Portugal rmartins.víseu@gmail.com; 2 Escola Superior de Saúde do Instituto Politécnico de Viseu, Portugal. E-mail: saraffgomes@hotmail.com Instituto Politécnico de Viseu Escola Superior de Saúde Instituto Politécnico de Viseu Portugal saraffgomes@hotmail.com; 3 Escola Superior de Saúde do Instituto Politécnico de Viseu, Portugal. Email: patricia.c.vicente@hotmail.com Instituto Politécnico de Viseu Escola Superior de Saúde Instituto Politécnico de Viseu Portugal patricia.c.vicente@hotmail.com; 4 Escola Superior de Saúde do Instituto Politécnico de Viseu, Portugal. Email: mnelia.carvalho@gmail.com Instituto Politécnico de Viseu Escola Superior de Saúde Instituto Politécnico de Viseu Portugal mnelia.carvalho@gmail.com; 5 Escola Superior de Saúde do Instituto Politécnico de Viseu, Portugal. Email: susanapbatista@gmail.com Instituto Politécnico de Viseu Escola Superior de Saúde Instituto Politécnico de Viseu Portugal susanapbatista@gmail.com

**Keywords:** Multiple Sclerosis, Quality of Life, Patients, Demyelinating Diseases, Central Nervous System, Esclerosis Múltiple, Calidad de Vida, Pacientes, Enfermedades Desmielinizantes, Sistema Nervioso Central, Esclerose Múltipla, Qualidade de Vida, Pacientes, Doenças Desmielinizantes, Sistema Nervoso Central

## Abstract

**Introduction:**

Multiple sclerosis significantly affects the quality of life of those suffering from this specific condition.

**Objective:**

To assess the quality of life of people with multiple sclerosis and analyse the correlation between the disease and its associated effects and different sociodemographic, clinical, and functional variables.

**Materials and Methods:**

An observational, cross-sectional, descriptive-correlational and quantitative study conducted using a non-probabilistic convenience sample composed of 70 patients suffering from multiple sclerosis registered with the Multiple Sclerosis Association of the Central Region of Portugal. The data collection protocol included sociodemographic and clinical questions, the Family Apgar Scale, and the Barthel Index. Descriptive and inferential statistics were used to process the data. Data collection took place between April and July 2021.

**Results:**

The majority of participants reported a moderate overall quality of life (M=51,78 ± 24,09). Higher scores were observed in the social relationships and environmental health domains, while lower scores were recorded for the physical domain. Better quality of life was found to be positively associated with being under 45 years old, having higher educational qualifications, living in functional families, and experiencing greater functional independence in activities of daily living.

**Discussion:**

The variables with the strongest association were those capable of influencing the physical and social domains. Those variables explained 59.00% and 53.00% of the variability.

**Conclusions:**

These results indicate that people with multiple sclerosis have a compromised quality of life, highlighting the need for new strategies focusing on early diagnosis and effective preventive interventions meant to improve quality of life across all its domains.

## Resumen

**Introducción:**

La esclerosis múltiple afecta significativamente la calidad de vida de las personas que padecen la enfermedad.

**Objetivo:**

Evaluar la calidad de vida de personas con esclerosis múltiple y analizar la asociación con variables sociodemográficas clínicas y funcionales.

**Materiales y Métodos:**

Estudio observacional, transversal, descriptivo-correlacional y cuantitativo que utilizó una muestra no probabilística por conveniencia, compuesta por 70 pacientes con esclerosis múltiple, registrados en la Asociación de Esclerosis Múltiple de la Región Central de Portugal. El protocolo de recolección de datos contuvo preguntas sociodemográficas y clínicas, la Escala de Apgar familiar y el Índice de Barthel. En el procesamiento de los datos se utilizó estadística descriptiva e inferencial y la recolección se realizó entre abril y julio de 2021.

**Resultados:**

La mayoría de los participantes presentan una calidad de vida general moderada (M=51,78 ± 24,09), con puntuaciones más altas en las relaciones sociales y dominios ambientales y menores en el dominio físico. Una mejor calidad de vida se asoció con una edad inferior a 45 años, mayor nivel educativo, familias funcionales y mayor independencia funcional en las actividades de la vida diaria.

**Discusión:**

Las variables con asociación más robusta son las que influyen en el dominio físico y en el dominio social, que explican, en total, el 59,00% y el 53,00% de su variabilidad.

**Conclusiones:**

Estos resultados indican que las personas con esclerosis múltiple tienen una calidad de vida comprometida, fortaleciendo la necesidad de implementar nuevas estrategias de diagnóstico precoz e intervenciones preventivas efectivas, potenciando una mejor calidad de vida, en todos los ámbitos que la constituyen.

## Resumo

**Introduçâo:**

A esclerose múltipla afeta de forma bastante significativa a qualidade de vida das pessoas portadoras da doença.

**Objetivo:**

Avaliar a qualidade de vida da pessoa com esclerose múltipla e analisar a associaçâo com variáveis sociodemográficas clínicas e funcionais.

**Materiais e Métodos:**

Estudo observacional, transversal, descritivo-correlacional e de cariz quantitativo que utilizou uma amostra do tipo nao probabilístico por conveniência, constituida por 70 doentes com esclerose múltipla, inscritas na Associaçâo de Esclerose Múltipla da Regiâo Centro de Portugal. O protocolo de recolha de dados continha questóes sociodemográficas, clinicas, a Escala de Apgar familiar e o Índice de Barthel. Foi utilizada estatística descritiva e inferencial no tratamento de dados e a colheita foi efetuada entre abril e julho de 2021.

**Resultados:**

A maioria dos participantes apresenta qualidade de vida global moderada (M=51,78 ± 24,09), com scores mais elevados nos dominios relaçôes sociais e ambiente e mais baixos no dominio físico. A melhor qualidade de vida estava associada, a idade inferior a 45anos, habilitaçôes literárias superiores, familias funcionais e maior independência funcional nas atividades de vida diária.

**Discussâo:**

As variáveis com associaçâo mais robusta sâo as que influenciam o domínio físico e o domínio social que explicam, no seu total, 59,00% e 53,00% da sua variabilidade.

**Conclusôes:**

Estes resultados indicam que as pessoas com esclerose múltipla apresentam uma qualidade de vida comprometida, fortalecendo a necessidade de implementar novas estratégias de diagnóstico precoce e intervençôes preventivas eficazes, potenciadoras de melhor qualidade de vida, em todos os domínios que a constituem.

## Introduction

Multiple sclerosis (MS) is an autoimmune, inflammatory and degenerative disease that affects the Central Nervous System^1^. It is a chronic, demyelinating, progressive and disabling disease with an unpredictable evolution that negatively affects the patients’ well-being and quality of life (QoL). It particularly disrupts myelin, an insulating sheath that surrounds, nourishes, protects, and electrically insulates the extensions of neurons, allowing electrical impulses to transmit quickly and efficiently^2^^, ^^3^.

MS is considered the most frequent non-traumatic cause of disability among young adults aged between 20 and 40. Studies have shown a higher incidence among women, with a ratio of 2.3:1. Relapsing-remitting MS is more frequently observed among women, but primary progressive MS (PPMS) has been found to be identically prevalent in both men and women^4^^, ^^7^. The influence of heredity is often a subject of debate. However, according to neurologist Filipe Palavra^8^, the risk of a person developing this disease in Portugal increases from 0.3% among the general population to between 1.5% and 3% if one of the parents has been diagnosed with the disease. Even so, the maximum genetic risk currently known is approximately 30% (in monozygotic twins), which leaves 70% of the risk determinants to environmental factors and/or factors of different origins^9^^, ^^10^.

As a chronic illness, MS involves restructuring and adapting to new lifestyles, and both the individuals and their families have to learn how to cope with this new situation. This adaptation depends on a wide range of factors that encompass individual characteristics, values, beliefs, cultural environment, acceptance, and life expectations^11^. In addition, the onset of the disease (particularly at an early stage in life) has a strong impact on future projects, education, social relationships, marriage, and family and professional life. Consequently, in this dynamic process, the illness of one of the members will soon become a family illness, affecting the overall QoL of the whole family^12^.

QoL is a multidimensional and subjective concept, defined by the World Health Organisation (WHO) as an individual's perception of their position in life in the context of the culture and value systems of the society in which they live and in relation to their goals, expectations, standards, and concerns^3^. It is a broad-ranging concept that explains the complexity of the construct, interrelating the environment with physical and psychological aspects, level of independence, social relationships, and personal beliefs.

Interest in the QoL of people with MS in Portugal is relatively recent since, for many years, the focus was merely placed on assessing the level of disability and impairment caused by this disease. The knowledge gained from nurses' daily practice shows that being diagnosed with MS does not preclude patients from having a good QoL. On the other hand, the constraints of the disease should not make the person feel less of a human being, citizen, family member, friend, or professional^13^.

The Multiple Sclerosis International Federation (MSIF) defined seven principles to improve QoL, ranging from patient empowerment, independence and their capacity to play a central role in decisions that affect their lives; access to comprehensive and effective treatment and care; support for the network of family, friends, loved ones and unpaid caregivers; accessible and flexible work, volunteering, education, and leisure opportunities; accessible public and private spaces, technological support and adapted transport; financial resources to meet the evolving needs and costs of living with MS; supportive attitudes, policies and practices that promote equality and challenge stigma and discrimination^14^^, ^^15^.

The assumptions described and the challenges posed by WHO, the Directorate General of Health (DGS), and the Portuguese Multiple Sclerosis Association (APEM) to minimise the consequences of MS and promote the patient's well-being and QoL led to the development of this study. Its primary aim was to assess the QoLof people with MS and analyse possible associations with sociodemographic and clinical variables.

## Materials and Methods

A cross-sectional, descriptive/relational quantitative study was conceptualised. The non-probability convenience sample consisted of 70 patients with confirmed MS and registered with the Multiple Sclerosis Association of the Centre of Portugal. In order to be eligible, participants had to meet the following inclusion criteria: being 18 years of age or older, having an established diagnosis of the disease, and having had the disease for at least three years. Exclusion criteria included various medical conditions, such as dementia and other associated degenerative diseases. A questionnaire was used as the data collection instrument (DCI). It was composed of three sections: the first covered the participants’ sociodemographic background (age, gender, marital status, academic qualifications, cohabitation, area of residence, and professional situation); the second included questions about the respondents’ clinical situation (at what age were they diagnosed with MS, what were the symptoms at the onset of the disease, what symptoms are patients currently experiencing, when did they have their last crisis, and what other associated disease are they suffering from) and the third included three scales already tested and validated for the Portuguese population: the Family APGAR Scale^16^, Barthel Index^17^ and the WHOQOL-BREF^18^.

The Family APGAR Scale is a 5-point Likert scale developed to quantify an individual's degree of satisfaction with their family's functioning. The scale overall score is obtained by summing the score given to each question and ranges from zero (0) to ten (10) points. Higher scores indicate greater satisfaction with family functionality. The Barthel Index is a scale that assesses the subject's level of independence in carrying out 10 basic activities of daily living (ADLs): feeding, bathing, grooming, dressing and undressing, bowel control, bladder control, toileting, ambulation, chair transfer, and stair climbing. The scale score ranges from 0 to 100 points. The lower the score, the more dependent the patient is in completing ADLs. The WHOQOL-BREF is a 26-item instrument. The questions included in this instrument focus on the individual’s overall perception of their health and quality of life. It also contains two general questions whose aim is to obtain information about the participants’ overall perception of their health and QoL. The items were formulated in strict accordance with specific methodologies suggested by WHO. Items are rated on a 5-point Likert scale. This self-administered questionnaire addresses four QoL domains: physical health, psychological health, social relationships and environmental health. Scores range from 0 to 100 points, with a cut-off point of 50. Scores above 50 correspond to better QoL, while scores below 50 correspond to a lower QoL.

Data was collected between April and July 2021. Before collecting the data, participants were asked to voluntarily collaborate in the study by signing an informed consent form. Information about the research objectives was provided (on the day of the medical consultation), and the confidentiality of the answers and clinical judgements made was ensured, in full compliance with the ethical and legal principles set out in the Declaration of Helsinki. In addition, the study received a favourable opinion from the Health Ethics Committee through Order N° 09/2021.

The data was processed using the Statistical Package for the Social Science programme (IBM® SPSS® Statistics) - version 25 and the SPSS Analysis of Moment Structures module (IBM® SPSS® Amos). Non-parametric tests were used for variables with non-normal distributions, whereas parametric tests were used for variables with normal distributions. The analysis included the estimation of at least one parameter and interval or proportion levels, in accordance with the metric and normality parameters required for their application. Student's t-tests, Chi-squared tests, adjusted residuals, U-Mann Whitney (UMW) tests, Kruskall-Wallis test, and multiple and univariate linear regressions were consequently used. As a complement, and given that more than two group means were tested for equality, post hoc tests were conducted to determine the differences observed between each group. The statistical tests used 95% confidence intervals and/or 5% significance levels. The data collected for this study is stored in the Mendeley Data repository^19^.

## Results

Participants ranged in age from 18 to 71, with a mean age of 49.80 years (± 12.77), and were mostly female (68.62%). The majority (51.38%) were married or in a non-marital partnership and had completed lower secondary education (the Portuguese 3rd cycle) (54.30%). In terms of family households, 57.14 % come from nuclear families, predominantly living in urban areas (68.62%) (see Table 1).

**Table 1:** Statistics related to age for both genders

Variables	Total (70)	Male (22)	Female (48)	p-value
Marital status Without a partner With a partner	48.62(34) 51.38(36)	72.71(16) 27.33(6)	37.51(18) 62.50(30)	0.075
Academic qualifications Lower secondary education High school Higher Education	54.30(38) 17.18(12) 28.52(20)	63.60(14) 27.31(6) 9.12(2)	50.00(24) 12.51(6) 37.50(18)	0.200
Family household Nuclear family Extended family Others	57.14(40) 31.45(22) 11.41(8)	36.45(8) 36.41(8) 27.32(6)	66.73(32) 29.26(14) 4.21(2)	0.061
Home area Urban Rural	68.62(48) 31.38(22)	54.51(12) 45.50(10)	75.00(36) 25.00(12)	0.263

*p-value: Fisher exact test*

Evidence shows that most of the participants (80.00%) were not working when they filled out the questionnaire. Among these, 83.30% were female and 72.71% were male.

Data on the participants' perceptions of family functioning (scores 0-10) shows that the average score for the total sample is 6.29± 3.13. Scores were higher among female (M=6.89± 2.89) than among male participants (M=5.45± 3.58). Findings also revealed that 57.13% of the respondents perceived their families as dysfunctional, while 42.90% perceived them as functional. Clinical data, on the other hand, revealed that, for most participants, the average age of MS diagnosis was 32.29 years old (±10.43). Evidence showed a wide range of early symptoms that included loss of balance and difficulties with coordination (62.93%), fatigue (54.31%), vision problems (48.61%), mood changes (45.74%), and spasticity (25.72%). At the time of data collection, the most prevalent symptoms were fatigue (80.00%), pain (71.40%), loss of balance (68.62%), problems with concentration and learning (51.41%), bladder control problems (42.90%), spasticity (40.00%), bowel control, and sexual disturbances (34.32%). When asked about the disease onset, 54.31% of the participants stated that it had occurred approximately 2 years ago, while 45.73% of the patients reported having had the disease for over 2 years.

Responses also indicated that many of these patients had other associated pathologies such as osteoporosis (11.41%), cardiovascular disease (5.72%), respiratory infections (3.95%) and kidney disease (2.91%).

The assessment of the functional capacity of patients with MS to carry out the basic ADLs revealed that 22.91% of the participants heavily depended on someone else to be able to perform those basic routines, 31.40% were partially dependent and 45.71% were totally independent. They required total/ partial support to engage in activities such as bathing, ambulation, stair climbing, and chair transfer. Male subjects were even more dependent than females, even though the statistical differences were not significant (p>0.05).

The study also focused on existing rehabilitation programmes and, in this regard, the primary aim was to assess the level of involvement of rehabilitation nurses in the process: the analysis conducted showed that only 34.32% of MS patients were accompanied by these nurse specialists, while the remaining 65.78% were accompanied by other healthcare professionals. The participants who had been followed by the RNs considered their interventions to be extremely important and believed that they greatly contributed to the promotion of their QoL (66.73%). This recognition was particularly pronounced among female patients. Participants also claimed (66.73%) that the intervention of the RNs differs from that of other professionals due to the holistic perspective they adopt when carrying out their work.

The results obtained show a moderate overall perception of QoL (M=51,78 ± 24,09) on a scale of 0-100 points (see Table 2). The analysis of the different domains demonstrates the existence of poor QoL in the physical (M=49,59 ± 14,48) and psychological (M=44,88 ± 13,78) domains, whereas the highest mean scores are associated with the social relationships (M=55,47 ± 15,64) and environmental health (M=53,48 ± 11,66) domains.

**Table 2:** QoL Statistics

Quality of Life	Min	Max	M ± SD
Physical domain	17.86	75.00	49.59 ± 14.48
Psychological domain	12.50	79.17	44.88 ± 13.78
Social relationships domain	25.00	83.33	55.47 ± 15.64
Environmental health domain	25.00	78.13	53.48 ± 11.66
Overall perception of QoL	12.50	100.00	51.78 ± 24.09

*M-Mean, SD-Standard Deviation*

The results related to the correlation between variables are presented below and include only the variablesinwhich statistically significantdifferences were found (p<0.05).That way,resultsdemonstrate that participants aged under 45 had better QoL but only in the physical (Z = 20,500 p=0.000) and social relationships domains (U =74. 000 p=0.024); higher academic qualifications (higher education) were also associated with better QoL in the physical (x^2^=11.122 p<0.033) and environmental health domains (x^2^=7.757 p<0.021); similarly, patients who perceived their families as functional had better QoL in the social relationships ^=86. 000 p<0.031), environmental health ^=51.500 p<0.001), and overall QoL domains ^=63.500 p<0.003). Participants who show greater independence in carrying out ADLs had better QoL in the physical (Z = 48,500 p = 0.001), psychological (Z = 81,000 p = 0.020) and social relationships domains (Z = 85,000 p = 0.029); better QoL was also found among patients who have not experienced any outbreak of the disease for more than 2 years, but only in the physical domain (t = 3.686 p = 0.012). Slight differences in the mean scores were found in some other variables (gender, marital status, occupational status, cohabitation, home area, age at diagnosis, and being followed by RNs), but they were not statistically significant (p>0.05).

**Figure 1:**
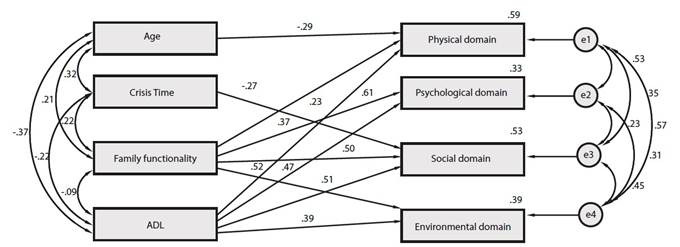
Final graphical output for the multivariate linear regression.

To assess predictive variables of the participants’ QoL, a multivariate linear regression model was constructed using the ‘stepwise’ estimation method. The final adjusted (refined) model is shown in Figure 1. A brief analysis of the model shows that the variables with the highest correlation weight are those which influence the physical and social domains. These variables explain 59% and 53.0% of the variability, respectively. A closer look at the specificity of the variables reveals that those with the lowest correlation weight were age (showing an inverse relationship) and the time elapsed since the last MS episode, while the highest correlations were observed in variables such as positive perceptions of family functionality and greater performance in ADLs.

## Discussion

The sociodemographic characteristics of the participants are in line with those provided by recent studies conducted with similar target populations^1^^, ^^4^^, ^^6^. The sample was mainly composed of female patients, with an average age of 49.80 years. Most were married and had completed lower secondary education (the Portuguese 3° Ciclo de Escolaridade)^6^. Evidence also showed that the majority of the patients came from nuclear families and resided predominantly in urban areas. Professionally, 80% of the participants were unemployed. The highest unemployment rate was observed among female patients.

These findings partially confirm the expected correlation with the statistical data published in Portugal by the Instituto Nacional de Estatistica^6^ (National Statistics Institute), Direçâo Geral de Saude^4^^)^ (Directorate General of Health) and the Portuguese Multiple Sclerosis Association^1^.

For Martins et al.^20^, the way families react to situations of chronic illness significantly affects the QoL of the patients, both positively and negatively. The authors claim that it all depends on whether or not family members possess the structural and psychological-emotional conditions required to embrace this new reality and on how the functionality of the family system is perceived^20^. This last variable caused a divide among the respondents since, in this study, 42.90% of the participants perceived their families as functional and 57.10% as dysfunctional. As highlighted by Lysandropoulos et al.^21^, this circumstance may adversely impact MS patients’ QoL, as it directly affects important and essential aspects of their lives, such as self-esteem and the ability to manage their own lives and all related activities^21^.

The clinical profile of the sample unveiled some common characteristics: the average age for MS diagnosis was 32.29 years and patients reported a wide range of early symptoms, such as loss of balance and difficulty with coordination, fatigue, vision problems, mood fluctuations, and spasticity. However, in a longitudinal assessment of the disease, fatigue, pain, loss of balance, problems with concentration and learning, bladder problems, spasticity, and sexual dysfunction emerged as the most prevalent symptoms, in that specific order. For most, the onset of the disease occurred two or more years ago and was accompanied by concomitant associated pathologies, particularly osteoporosis, cardiovascular disease, respiratory infection, and kidney disease. These findings are in line with the studies conducted by Lysandropoulos et al.^21^, Manjaly et al.^22^ and Santos^23^ that also highlight the pivotal role played by rehabilitation nurses, both in preventing complications and managing respiratory functionality.

The analysis of the functional capacity of MS patients to carry out basic ADLs revealed several differentiated stages and situations: the majority of participants exhibited some degree of dependence (total or partial) when carrying out these activities and required assistance with activities such as bathing, stair climbing, chair transfers, and ambulation. Evidence also showed that the levels of dependence were higher among male subjects compared to female subjects.

These results largely corroborate findings from other studies^23^^, ^^24^ and highlight the importance of implementing rehabilitation nursing programmes that focus essentially on optimising self-care in ADLs and IADLs, encouraging the implementation of mobilisation exercises for the different body segments, improving ambulation and balance and preventing fall, reducing patients’ dependency, and managing symptoms to improve QoL^25^^, ^^26^.

The benefits of specific interventions provided to patients with chronic illnesses by rehabilitation nurses are widely recognised. However, in this sample, their visibility is still quite incipient, as only a third of the patients were followed by these specialised professionals. This proves that the provision of services (inpatient and community-based) supported by professionals who possess the specialised skills to manage these patients’ condition appropriately is urgently required. The patients’ assessment of the rehabilitation nurses’ performance is quite positive. The way they differentiate themselves from other health care professionals, the holistic perspective they adopt in their work, and their focus on promoting QoL are the aspects most commonly highlighted.

There is currently a substantial number of robust and convergent studies focusing on the variability of QoL levels in MS patients. The perception of QoL (overall score) found in this study is moderate. Lower scores are observed in the psychological and physical domains, while the highest mean values are associated with the social relationships and environmental health domains. Several studies have reported a relationship between emotional distress and MS, as these patients often exhibit depressive symptoms^22^^, ^^27^^, ^^28^. Depression is actually a reaction to the disease, which, due to its characteristics, is highly stressful and can adversely affect the most diverse areas of an individual's life, namely, family dynamics, social relationships, professional status, and physical independence, among others. On the other hand, the improvements observed in the social relationships and environmental health domains can be explained not only by the resilience developed by the patients, who progressively manage to adapt to the diagnosis and the psychological, physical, and social demands associated with the treatments, but also to the different measures that have gradually been implemented to promote QoL.

The analysis of the correlation between variables showed that participants aged under 45 demonstrated better QoL, but only in the physical and social relationships domains; higher levels of education were also associated with better QoL, both in the physical and environmental health domains; similarly, patients who perceived their family as functional seemed to experience better QoL in the social relationships, environmental health, and overall QoL domains; patients who were more independent in carrying out ADLs reported better QoL in the physical, psychological and social relationships domains; and finally, patients whose last disease outbreak had occurred less than 2 years ago also exhibited better QoL, but only in the physical domain.

The existing literature^5^^, ^^8^^, ^^14^ consistently shows that people with MS tend to experience a lower QoL compared to patients suffering from other pathologies. For those patients, a lower QoL is observed across all health-related QoL domains. Despite the unique evolution of each case and the existence of treatments that make it possible for the patient to lead a normal life, maintain their employment, engage in social activities, or start a family, MS is a condition characterised by a progressive disability that inevitably affects the patients’ physical and psychological performance, social relationships, and overall QoL. Some psychosocial predictors associated with people with chronic illnesses identified so far include greater socio-emotional support, lower subjective burden, a higher quality relationship with the patient and greater intrinsic motivation for self-care^15^^, ^^29^. These findings are partially corroborated by this study since the variables which best explain the QoL dimensions of people with MS are the ability to perform ADLs and a positive perception of family functionality. These variables have greater predictive weight in the physical and social domains^30^^, ^^31^.

The QoL observed in this study is clearly multidimensional and largely aligns with the results reported by other authors^12^^, ^^27^^, ^^31^. However, it diverges from other research conducted on MS patients in Portugal, both in terms of the sample’s sociocultural and family specificities and in terms of the value attached to the QoL dimensions. The recent emphasis placed on the basic and advanced training of nurses in "holistic care" seems to be paying off.

QoL is a broad, complex construct that encompasses objective and subjective dimensions and addresses all aspects of human life (physical, emotional, social, and environmental). This complexity is evident across all the dimensions addressed by the young adults who composed the sample, with particular emphasis placed on the social relationships and environmental health domains, two processes that have only recently been incorporated into conceptual and practical models used to design health promotion policies and strategies. This broadening and strengthening of intersectoral approaches to health policies and actions provide a greater understanding of these conceptions and will support the planning of educational campaigns meant to foster better and more efficient self care and fulfilment practices among these populations.

Although we are aware of the limitations of this study, considering both the size and sample typology (which impede the generalization of results) and the data collection methodology (a questionnaire, which may give rise to subjective perceptions), we believe that the information provided can prove valuable for healthcare professionals, especially rehabilitation nurses. The study offers insights into the conceptions of health and chronic illness that are pivotal to the development of patient intervention programmes designed to improve MS patients’ QoL. We recommend that healthcare professionals continue to study the subject but using more robust samples and longitudinal methods.

## Conclusion

This study has demonstrated that people with MS in Portugal typically experience a moderate (overall) QoL and that the lowest scores were observed in the physical and psychological domains. Conversely, the dimensions related to social relationships and environmental health are highly valued and obtained the highest scores. This positive perception may have been influenced by the development of various initiatives led by policy makers, healthcare professionals, researchers, and economic agents, all of which deeply contributed to fostering continuous and widespread improvement in QoL for people with MS.

A better understanding of how people with MS assess their QoL and its increased interaction with the different domains (especially associated variables) will provide crucial insights for planning and implementing nursing interventions aimed at optimising these patients' QoL.

The regular care provided to patients should involve a comprehensive and multidisciplinary approach that will necessarily include education and guidance, clinical monitoring, comparative functional assessment, symptom management, self-management training, and sustained information on advances in research and new treatment options.

## References

[1] Sociedade Portuguesa de Esclerose Múltipla (2021). Arquivos sobre Esclerose múltipla.

[2] Thompson AJ, Banwell BL, Barkhof F, Carrol WM, Coetzee T, Comi G (2018). Diagnosis of multiple sclerosis:2017 revisions of the McDonald criteria. Lancet Neurol.

[3] World Health Organization (2008). Atlas multiple sclerosis resources in the world.

[4] Direçâo Geral de Saúde (2022). Organizaçâo de Cuidados na Esclerose Múltipla.

[5] Rente CPS, Ferreira JAS, Garrett ACM (2017). Qualidade de vida da pessoa com esclerose múltipla e dos seus cuidadores. Revista de Enfermagem Referência.

[6] Instituto Nacional de Estatística (2019). Estimativas de populaçâo residente em Portugal.

[7] Fernandez O (2018). Is there a change of paradigm towards more effective treatment early in the course of apparent high-risk MS?. MultScler Relat Disord.

[8] Gomes I, Ribeiro J, Palavra F (2022). Monitoring and Managing Patients with Tuberous Sclerosis Complex: Current State of Knowledge. Journal of Multidisciplinary Healthcare.

[9] Marques VD, Passos GR, Mendes MF, Callegaro D, Lana-Peixoto MA, Comini-Frota ER (2018). Brazilian consensus for the treatment of multiple sclerosis: Brazilian Academy of Neurology and Brazilian Committee on Treatment and Research in Multiple Sclerosis. Arq Neuropsiquiatr.

[10] Baumstarck K, Pelletier J, Boucekine M, Auquier P, Group MusiQoL Study (2015). Predictors of quality of life in patients with relapsing-remitting multiple sclerosis: a 2-year longitudinal study. Rev Neurol.

[11] Brownlee WJ, Hardy TA, Fazekas F, Miller DH (2017). Diagnosis of multiple sclerosis: progress and challenges. Lancet.

[12] Nogueira IC, Porto ACP, Silva EG, Melo PO, Belchior LD, Loiola LMCV (2017). Avaliaçâo da ansiedade, depressao e qualidade de vida em pacientes com esclerose múltipla. Rev Inspirar Mov Saúde.

[13] Morrow SA (2018). Anxiety is more important than depression in MS - Yes. MultScler J.

[14] Federaçâo Internacional da Esclerose Múltipla (2021). Dia Mundial da Esclerose Múltipla: entenda a doença e seus sintomas.

[15] Ysrraelit MC, Fiol MP, Gaitán MI, Correale J (2018). Quality of life assessment in multiple sclerosis: different perception between patients and neurologists. Front Neurol.

[16] Andrade A, Martins R (2016). Funcionalidade Familiar e Qualidade de Vida dos Idosos. Rev. Mill.

[17] Cid-Ruzafa J, Damián-Moreno J (1997). Valoración de la discapacidad física: el índice de Barthel. Rev. Esp. Salud Publica.

[18] WHOQOL Group (2008). The World Health Organization Quality of Life Assessment (WHOQOL): Development and general psychometric properties. Social Science e Medicine.

[19] Martins R, Gomes S, Vicente P, Carvalho N, Batista S (2024). Coniunto de dados: A qualidade de vida de pessoas com esclerose múltipla em Portugal. Mendeley.

[20] Martins R, Santos C (2020). Capacitaçâo do cuidador informal: O papel dos enfermeiros no processo de gestao da doença. Gestâo e Desenvolvimento.

[21] Lysandropoulos AP, Havrdova E (2015). 'Hidden' factors influencing quality of life in patients with multiple sclerosis. Eur J Neurol.

[22] Manjaly ZM, Harrison NA, Critchley HD, Do CT, Stefanics G, Wenderoth N. (2019). Pathophysiological and cognitive mechanisms of fatigue in multiple sclerosis. J NeurolNeurosurg Psychiatry.

[23] Santos M, Sousa C, Pereira M, Pereira MG (2019). Quality of life in patients with multiple sclerosis: A study with patients and caregivers. Disabil Health J.

[24] Casetta I, Riise T, Nortvedt MW, Economou NT, De Gennaro R, Fazio P (2019). Gender differences in health-related quality of life in multiple sclerosis. Multiple Sclerosis.

[25] Regulamento n.° 125/ (2011). Regulamento das competências específicas do enfermeiro especialista em enfermagem de reabilitaçao. Diário da República.

[26] Pereira LD, Bellinati NVC, Kanan LA (2018). Self-Efficacy for Managing Chronic Disease 6-Item Scale: avaliaçâo da autoeficácia no gerenciamento da doença crónica. Rev Cuid.

[27] Morales RR, Morales NMO, Rocha FCG, Fenelon SB, Pinto RMC, Silva CHM (2007). Qualidade de vida em portadores de esclerose múltipla. Arq. Neuro-Psiquiatria.

[28] Alhazzani AA, Alqahtani MS, Ogran H, Abuhawi OH, Asiri AY, Al-Hanash AM (2018). Depression severity and its predictors among multiple sclerosis patients in Saudi Arabia: a cross-sectional study. NeuroimmunolNeuroinflammation.

[29] Páez Esteban AN, Torres Contreras CC, Campos de Aldana MS, Solano Aguilar S., Quintero Lozano N., Chaparro Díaz OL (2020). Custos diretos e indiretos do cuidado de pacientes com doenças crónicas näo transmissíveis. Aquichan.

[30] Almeida F, Martins R, Martins C (2022). Capacitaçâo do cuidador Informal: estudo das dificuldades e das variáveis preditivas. Investigación en enfermería: Imagen y Desarrollo.

[31] Tauil CB, Grippe TC, Dias RM, Dias-Carneiro RPC, Carneiro NM, Aguilar ACR (2018). Suicidal ideation, anxiety, and depression in patients with multiple sclerosis. Arq Neuropsiquiatr.

